# Extensive Arteriovenous Fistula Thrombosis With Glucagon-Like Peptide-1 Agonist

**DOI:** 10.7759/cureus.88015

**Published:** 2025-07-15

**Authors:** Muhammad Muneeb, Elizabeth Choi, Coralys Rodriguez, Kanika Goyal, Zainab Bhura, Ghadeer Hannoudi

**Affiliations:** 1 Internal Medicine, Trinity Health Oakland/Wayne State University, Pontiac, USA; 2 Internal Medicine, Wayne State University School of Medicine, Detroit, USA; 3 Internal Medicine, Ross University School of Medicine, Bridgetown, BRB; 4 Hematology and Oncology, Barbara Ann Karmanos Cancer Institute/Wayne State University, Detroit, USA; 5 Faculty of Medicine, Trinity Health Oakland/Wayne State University, Pontiac, USA

**Keywords:** av fistula thrombosis, deep vein thrombosis, glp-1 agonist, renal cell carcinoma (rcc), semaglutide

## Abstract

Glucagon-like peptide-1 (GLP-1) receptor agonists are widely used due to their significant anti-atherosclerotic, cardiovascular, and renoprotective benefits. However, potential adverse effects must also be considered. Here, we present a rare case of GLP-1 receptor agonist-induced thrombotic complications in a middle-aged African American female, contributing to the limited existing literature on this topic. A 55-year-old African American female with a past medical history of deceased donor renal transplantation presented with pain, swelling, and erythema localized to the left arteriovenous (AV) fistula. She also reported fever, chills, and the absence of a palpable thrill over the fistula for approximately one week before presentation. On physical examination, she exhibited significant swelling of the left upper limb with no detectable thrill over the AV fistula. Venous and arterial Doppler ultrasound of the left upper extremity revealed extensive thrombosis involving the left AV fistula, brachiocephalic vein, and both the radial and ulnar arteries. An extensive prothrombotic workup, including evaluations for inherited and acquired hypercoagulable states, was unremarkable. The only notable change in her medical regimen was a recent dose escalation of her GLP-1 receptor agonist. Given the temporal association and absence of alternative causes, the thrombosis was attributed to the GLP-1 receptor agonist, which was subsequently discontinued. No further thrombotic events occurred during follow-up. This case highlights one of the rare thrombotic events of commonly used drugs, such as GLP-1 receptor agonists. This case also underscores the importance of life-threatening events of commonly used drugs, adding to the existing literature.

## Introduction

Arteriovenous (AV) fistula thrombosis is one of the most common complications in patients undergoing long-term hemodialysis [1-3]. The development of such thromboses is attributed to the following factors: impaired venous outflow, inadequate arterial inflow, or predisposing conditions such as a hypercoagulable state or atherosclerotic cardiovascular disease [2]. Identified risk factors for AV fistula thrombosis include older age, female sex, elevated C-reactive protein (CRP) levels, and distal access sites [3]. In general, other conditions that increase the risk of thrombosis include the use of certain medications (e.g., immunosuppressive therapy), prolonged immobilization or hospitalization, malignancy, pregnancy, and diabetes mellitus [4-6].

Here, we describe a case involving a 55-year-old African American female with a history of deceased donor renal transplantation who presented with a six-day history of pain, swelling, and erythema over her left AV fistula, accompanied by loss of thrill. Duplex ultrasonography revealed extensive thrombosis within the AV fistula, with extension into the left radial artery and evidence of deep vein thrombosis (DVT) in the left brachiocephalic vein. No abscess formation was identified. While her medical history and comorbidities may have contributed to a prothrombotic state, the recent dose escalation from 0.5 mg to 2 mg of semaglutide, a glucagon-like peptide-1 (GLP-1) receptor agonist, was considered a significant contributing factor to the thrombotic event.

## Case presentation

A 55-year-old African American female presented to the hospital with a one-week history of pain, swelling, and erythema localized to the AV fistula in her left arm. These symptoms were preceded by three to four episodes of diarrhea per day for a week, for which she had been hospitalized and treated with bowel rest and intravenous fluids.

Her past medical history was significant for end-stage renal disease secondary to hypertensive nephrosclerosis, for which she underwent a deceased donor renal transplant five years prior. The transplant was a four-antigen match, with alemtuzumab used for induction therapy. The patient had a functioning left upper extremity AV fistula created in 2010, which had not been in use since her transplant in 2019. Other known medical conditions include post-transplant diabetes mellitus, hypertension, and BK viremia. She had no known history of thrombosis or DVT. Her home medications included labetalol 200 mg twice daily, mycophenolate 500 mg twice daily, tacrolimus 3 mg daily, cinacalcet 30 mg daily, sodium bicarbonate 650 mg twice daily, and ferrous sulfate. She was also on semaglutide for weight loss, initiated five months prior at 0.25 mg and titrated up to 2 mg one month before presentation.

On presentation, the patient’s temperature was 101.3°F, blood pressure was 154/88 mmHg, heart rate was 124 beats per minute, and respiratory rate was 16 breaths per minute. The patient was alert, in no acute distress, nontoxic in appearance, and exhibited tachycardia with a regular rhythm. Her body mass index (BMI) was 36.67 kg/m². Local examination of the left upper extremity revealed swelling and erythema over the AV fistula, primarily in the antecubital region. The area was warm and tender on palpation, and no thrill was appreciated over the fistula.

Laboratory evaluation revealed a white blood cell count of 13.3 × 10⁹/L, hemoglobin of 10.7 g/dL, hematocrit of 34.5%, mean corpuscular volume of 89.4 fL, and platelet count of 433 × 10⁹/L. Renal function was stable with a creatinine of 1.14 mg/dL, blood urea nitrogen of 17 mg/dL, and an estimated glomerular filtration rate of 57 mL/minute/1.73 m² (Table 1). Coagulation studies revealed a prothrombin time of 16.1 seconds, an international normalized ratio of 1.4, and a partial thromboplastin time of 29.1 seconds (Table 2). Hemoglobin A1c was 6.6%.

**Table 1:** Complete blood count.

Laboratory test (unit)	Day of admission	Admission day 3	Day of discharge	Normal values
White blood cell count (K/µL)	13.3	10.4	11.6	3.7–11.0
Red blood cell count (K/µL)	3.86	3.08	3.11	3.8–5.2
Hemoglobin (g/dL)	11	8.7	8.7	12.0–16.0
Hematocrit (%)	34.5	27.4	27.7	35.0–46.0
Mean corpuscular volume (fL)	89.4	89	89.1	80.0–99.0
Mean corpuscular hemoglobin concentration (g/dL)	31.9	31.8	31.4	29.0–37.5
Red cell distribution width (%)	13.4	13.3	13.5	12.0–15.0
Platelets (K/µL)	433	420	470	150–450
Prothrombin time (seconds)	16.1	-	-	9.4–12.5
International normalized ratio	1.4	-	-	<4.5
Partial thromboplastin time (seconds)	29.1	-	-	25.1–36.5

**Table 2:** Procoagulant workup.

Laboratory test (unit)	Results	Normal values
Beta-2 glycoprotein 1 IgA antibody (U/mL)	1.9	<7.0
Beta-2 glycoprotein 1 IgG antibody (U/mL)	0.8 <2.4	<7.0
Beta-2 glycoprotein IgM antibody (U/mL)	65	<7.0
Protein S activity (%)	124	65–140%
Protein C activity (%)	2.2	70–135%
Cardiolipin IgA antibody	0.6	<14.0
Cardiolipin IgG antibody, cardiolipin IgM antibody	2.6	<10.0
Factor V Leiden	Negative	<10.0

**Figure 1:**
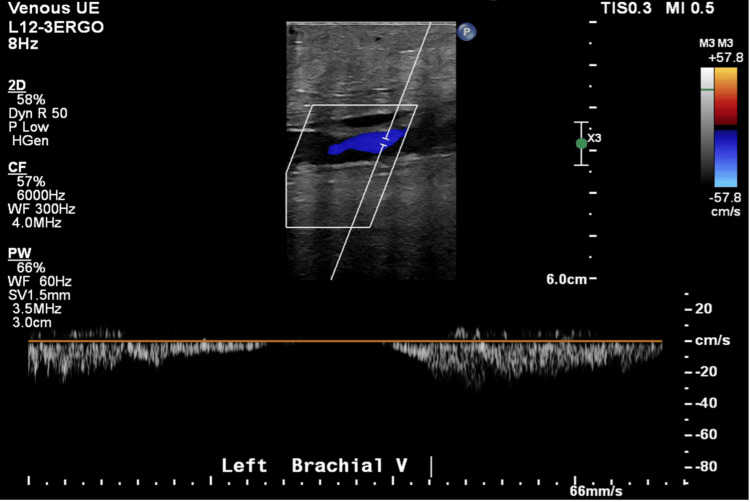
Left brachial vein duplex ultrasound.

**Figure 2:**
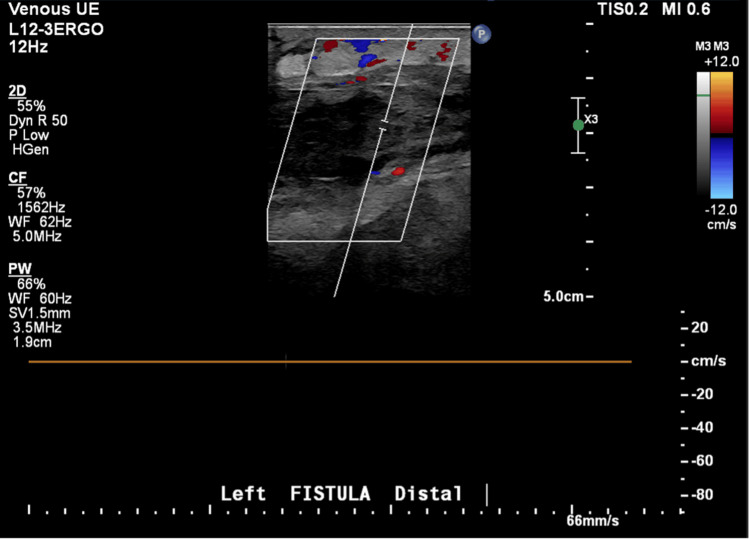
Left upper extremity venous duplex ultrasound.

**Figure 3:**
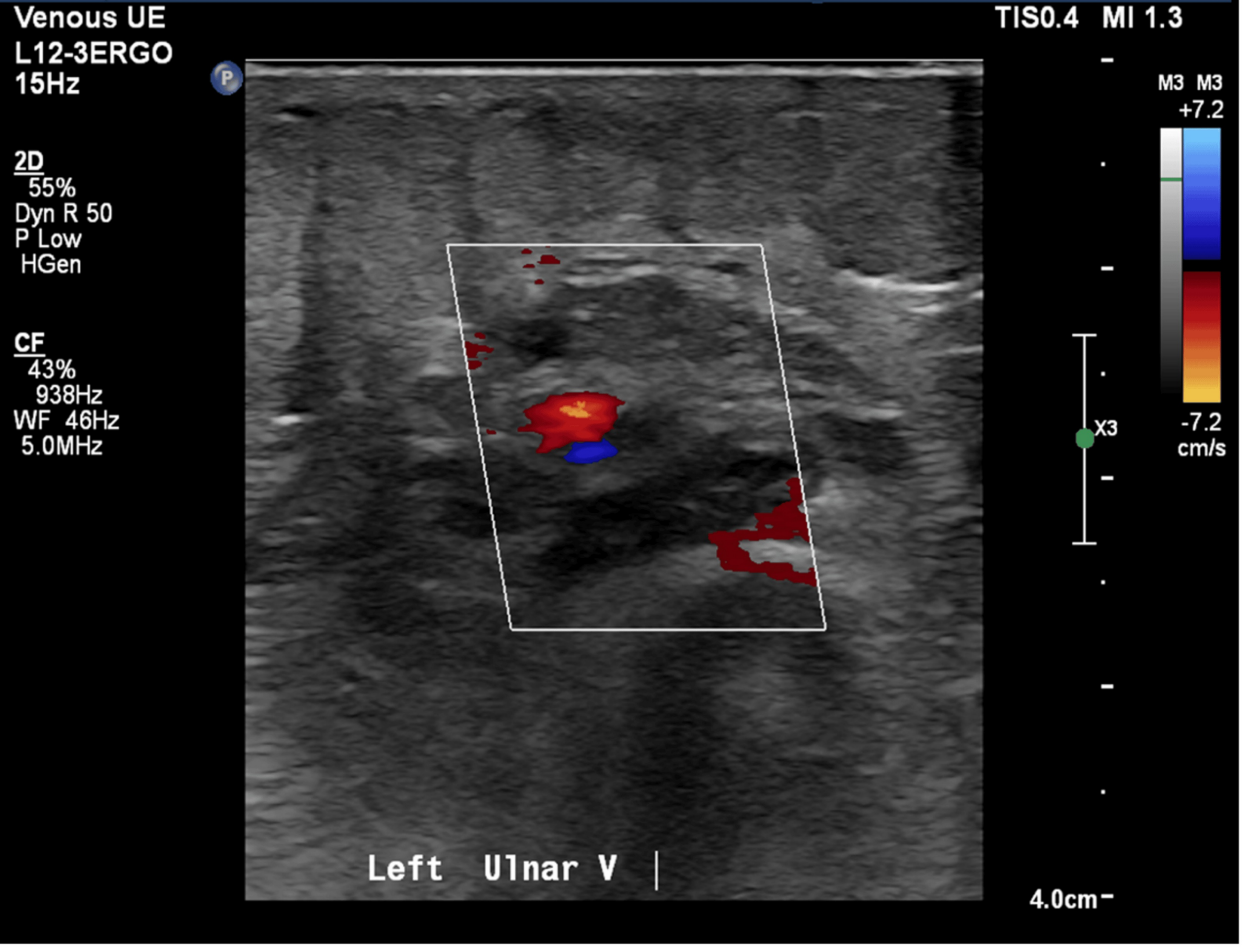
Left ulnar vein duplex ultrasound.

**Figure 4:**
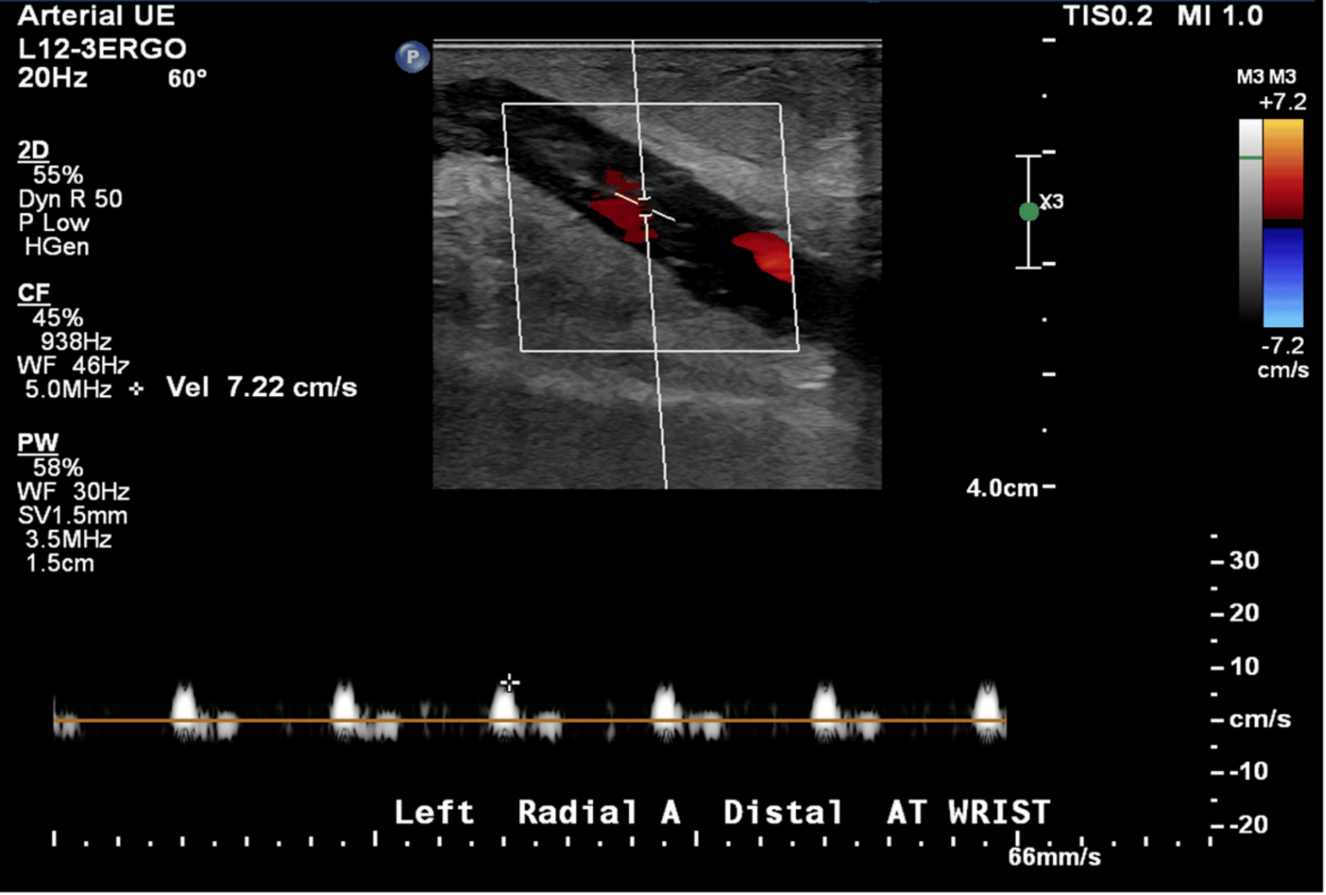
Left arterial upper extremity duplex ultrasound.

An ECG demonstrated sinus tachycardia. Vascular duplex ultrasound of the left upper extremity revealed extensive thrombosis throughout the AV fistula with extension into the left radial artery, as well as occlusive DVT in the left brachiocephalic vein (Figures 1-4). A soft tissue ultrasound of the left upper extremity showed diffuse soft tissue edema but no discrete fluid collection to suggest abscess formation.

A transthoracic echocardiogram revealed a normal left ventricular cavity size and wall thickness. Systolic function was hyperdynamic, with an ejection fraction of 72%. There were no regional left ventricular wall motion abnormalities or diastolic dysfunction, and there was no evidence of a left ventricular clot. A comprehensive procoagulant workup was negative.

Initial treatment for cellulitis of the left forearm included vancomycin 750 mg every 12 hours and ceftriaxone 1 g daily for five days. Blood cultures were sterile, ruling out bacteremia. Regarding the AV fistula thrombosis, the vascular surgery team was consulted, and it was determined that there was no indication for surgical intervention. The patient was placed on intravenous heparin at a rate of 20 units/kg/hour, with ongoing thrombotic workup.

The patient’s condition was stabilized on day two of admission, and her symptoms began to improve. The patient was discharged after five days of hospitalization on oral doxycycline for 10 days and oral apixaban. The patient followed up with Nephrology one month later with stable kidney function. She was followed up by her transplant team, and semaglutide was discontinued. A full prothrombotic evaluation remained unremarkable, and a follow-up visit with her primary care physician was arranged.

## Discussion

Diabetes mellitus is recognized as a substantial public health concern both in the United States and worldwide, with an estimated diabetic population of 643 million worldwide in 2030, and around 30-40% of diabetics experiencing renal disease [7,8]. Increasing need and interest in diabetic medications may reveal other potential modulators of thrombotic disease in patients with kidney disease.

GLP-1 receptor agonists, such as semaglutide, have gained widespread use in the management of type 2 diabetes due to their glycemic control, weight reduction, and cardioprotective benefits. Between January 2021 and December 2023, semaglutide prescriptions in the United States increased by 442%, from 471,876 to over 2.5 million fills, with a peak of nearly 2 million prescriptions in August 2023, a 392% increase from January 2021 [9]. While there may be adverse effects of semaglutide, it has shown great benefits in reducing systolic blood pressure, preventing major cardiovascular events, heart failure hospitalization, cardiovascular deaths, and stroke [10,11].

Recent case reports have documented instances of venous thromboembolism (VTE) in patients receiving GLP-1 receptor agonists for weight loss. These include portal vein thrombosis associated with semaglutide use [12], superior mesenteric vein thrombosis reported in patients after dose escalation of dulaglutide [13], and a case of DVT in a young man shortly after initiating tirzepatide therapy [14]. While these reports raise concern about a possible link between GLP-1 receptor agonists and VTE, especially in the context of weight loss treatment, they do not establish a causal relationship. Nevertheless, they provide preliminary evidence that warrants further investigation.

Interestingly, it had been previously proposed that GLP-1 agonists may play a role in reducing thrombus formation by activating endothelial nitric oxide synthase and increasing nitric oxide levels in platelets, which would decrease platelet activation and ultimately help prevent thrombosis [15]. Cameron-Vendrig et al. (2016) demonstrated that in vitro incubation of human megakaryocyte cells with GLP-1 agonist exenatide impaired thrombin, ADP, and collagen-mediated platelet aggregation [16].

**Figure 5:**
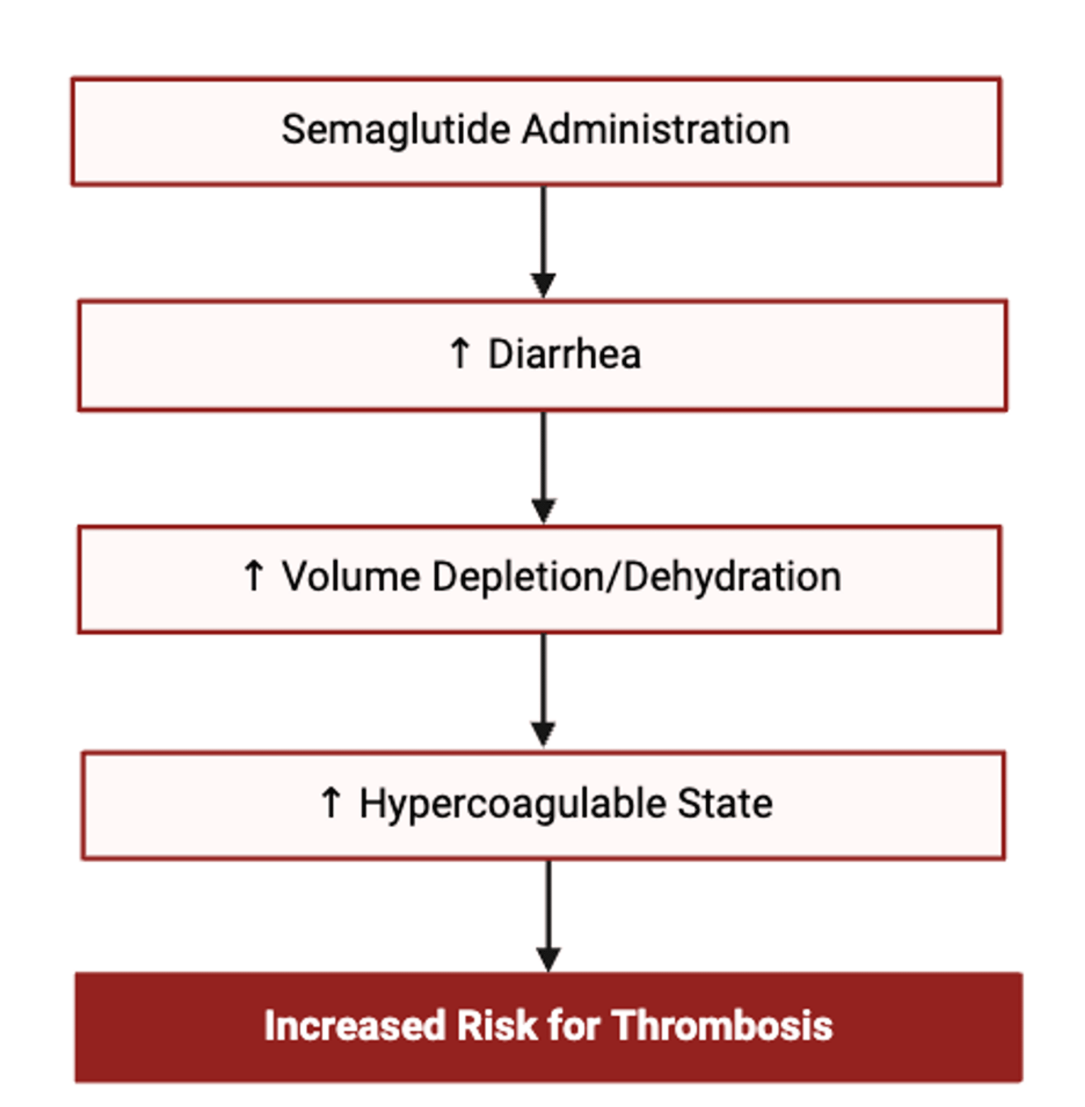
Proposed mechanism of the association between glucagon-like peptide-1 agonist and increased risk of thrombosis. [17].

However, emerging evidence complicates the narrative. A recent meta-analysis by Yin et al. (2021) reported a 266% increased risk of DVT associated with semaglutide use compared to control groups, despite an overall reduction in serious adverse events and a neutral impact on renal outcomes. The authors hypothesized that semaglutide-associated diarrhea may lead to dehydration, hemoconcentration, and increased blood viscosity, thereby contributing to thrombus formation [17]. In our case, a similar mechanism may have been at play. The patient experienced a week of diarrhea and dehydration shortly before the thrombotic event. The mechanism has been outlined in Figure 5.

With limited resources and minimal publications addressing this issue, the complexity of establishing causality in this case is evident. Temporality did correlate with the event; however, whether GLP-1 agonists attenuate platelet aggregation by altering cellular mechanisms or increase the risk of thrombus formation by inducing a dehydrated status in the context of the patient’s age, sex, and immunocompromised status, the increase in the patient’s semaglutide dose was likely one of many contributing factors that led to her extensive fistula thrombosis. This case highlights the urgent need for further investigation into the thrombotic risks associated with GLP-1 receptor agonists.

## Conclusions

Despite the potential risk of thrombosis associated with semaglutide, the therapeutic benefits may outweigh this risk. However, this case highlights a possible thrombotic complication in the setting of semaglutide use, whether due to rapid weight loss or dehydration from gastrointestinal side effects. Given the increasing use of GLP-1 receptor agonists, clinicians should be mindful to communicate this uncertainty to patients before starting treatment, especially in a population that is more susceptible to volume depletion. This case contributes to the limited existing literature and underscores the need for vigilance and individualized risk assessment when prescribing these agents.

## References

[1] Polkinghorne KR, Chin GK, MacGinley RJ (2013). KHA-CARI Guideline: vascular access - central venous catheters, arteriovenous fistulae and arteriovenous grafts. Nephrology (Carlton).

[2] Arasu R, Jegatheesan D, Sivakumaran Y (2022). Overview of hemodialysis access and assessment. Can Fam Physician.

[3] Zhang Y, Yi J, Zhang R, Peng Y, Dong J, Sha L (2022). Risk factors for arteriovenous fistula thrombus development: a systematic review and meta-analysis. Kidney Blood Press Res.

[4] Verhave JC, Tagalakis V, Suissa S, Madore F, Hébert MJ, Cardinal H (2014). The risk of thromboembolic events in kidney transplant patients. Kidney Int.

[5] McLendon K, Goyal A, Attia M (2025). Deep Venous Thrombosis Risk Factors. StatPearls.

[6] Vazzana N, Ranalli P, Cuccurullo C, Davì G (2012). Diabetes mellitus and thrombosis. Thromb Res.

[7] Gupta S, Dominguez M, Golestaneh L (2023). Diabetic kidney disease: an update. Med Clin North Am.

[8] International Diabetes Federation (2025). International Diabetes Federation. Diabetes now affects one in 10 adults worldwide. https://idf.org/news/diabetes-now-affects-one-in-10-adults-worldwide/.

[9] Scannell C, Romley J, Myerson R, Goldman D, Qato DM (2024). Prescription fills for semaglutide products by payment method. JAMA Health Forum.

[10] Verma S, Colhoun HM, Dicker D (2024). Semaglutide effects on cardiovascular outcomes in people with overweight or obesity (SELECT): outcomes by sex. J Am Coll Cardiol.

[11] Strain WD, Frenkel O, James MA (2022). Effects of semaglutide on stroke subtypes in type 2 diabetes: post hoc analysis of the randomized SUSTAIN 6 and PIONEER 6. Stroke.

[12] Farooqi MF, Khan M, Muhammad AM, Agha A (2025). Portal vein thrombosis in a patient on semaglutide. Qatar Med J.

[13] Manuel SL, Lin F, Kutty SM (2023). An atypical presentation of dulaglutide-induced pancreatitis complicated by superior mesenteric vein thrombosis. Cureus.

[14] Farooqi MF, Mehmood MA, Khan M, Salman HM, Agha A (2024). Extensive deep vein thrombosis in a young man taking tirzepatide for weight loss. AACE Clin Case Rep.

[15] Jia G, Aroor AR, Sowers JR (2016). Glucagon-like peptide 1 receptor activation and platelet function: beyond glycemic control. Diabetes.

[16] Cameron-Vendrig A, Reheman A, Siraj MA (2016). Glucagon-like peptide 1 receptor activation attenuates platelet aggregation and thrombosis. Diabetes.

[17] Yin DG, Ding LL, Zhou HR, Qiu M, Duan XY (2021). Comprehensive analysis of the safety of semaglutide in type 2 diabetes: a meta-analysis of the SUSTAIN and PIONEER trials. Endocr J.

